# Reliable enough to guide care? An umbrella review of hip arthroscopy meta‐analyses 2020–2025

**DOI:** 10.1002/jeo2.70640

**Published:** 2026-01-19

**Authors:** Nikolai Ramadanov, Maximilian Heinz, Maximilian Voss, Robert Prill, Roland Becker, Ingo J. Banke

**Affiliations:** ^1^ Center of Orthopaedics and Traumatology, Brandenburg Medical School University Hospital Brandenburg an der Havel Brandenburg an der Havel Germany; ^2^ Faculty of Health Science Brandenburg Brandenburg Medical School Theodor Fontane Brandenburg an der Havel Germany; ^3^ Clinic of Orthopaedics and Sports Orthopaedics, School of Medicine and Health, TUM University Hospital Technical University of Munich Munich Germany; ^4^ AGA‐Society for Arthroscopy and Joint‐Surgery Hip Committee, c/o Walder Wyss Ltd. Zürich Switzerland

**Keywords:** hip arthroscopy, meta‐analysis, methodological quality, patient‐related outcomes, umbrella review

## Abstract

**Purpose:**

Hip arthroscopy (HAS) evidence has expanded rapidly, but methodological quality and conclusions vary. This umbrella review of contemporary meta‐analyses published January 2020–October 2025 aimed to (i) identify eligible reviews, (ii) appraise methodological quality (AMSTAR 2) and review‐level risk of bias (ROBIS), (iii) quantify evidence overlap (corrected covered area, CCA) and (iv) map concordance of conclusions.

**Methods:**

We searched PubMed/MEDLINE, Embase and Epistemonikos (2020–2025) for human HAS meta‐analyses with quantitative synthesis on clinical effectiveness and safety. Two reviewers independently screened records and extracted data (consensus/third‐reviewer adjudication). Quality was assessed with AMSTAR 2, risk of bias with ROBIS, and evidence overlap with CCA. No re‐pooling of primary data.

**Results:**

From 5940 records, 44 meta‐analyses were included. AMSTAR 2 confidence was predominantly weak (≈7% high, 5% moderate; most critically low); ROBIS was low risk in just over half. Overlap was slight (low redundancy). Across randomised/comparative syntheses, HAS yielded superior short‐term improvements versus best‐practice nonoperative care—most consistently for iHOT‐33 at ~8–12 months—attenuating by ~24 months and not uniformly meeting MCIDs. Limited long‐term data suggest less radiographic osteoarthritis progression versus nonoperative care. Versus open procedures, functional outcomes were similar with fewer complications after HAS. Return‐to‐work was ~71% at ~115 days, return‐to‐sport was high (elite ~94% at ~6–7 months; broader cohorts ~89%). More recent evidence increasingly favours capsular closure and labral repair. Preoperative intra‐articular injection ≤3 months before HAS was associated with higher infection risk.

**Conclusion:**

Evidence supports short‐term benefits and a good safety profile for HAS, yet certainty remains limited. Prioritise patient selection and standardised rehab; high‐quality long‐term studies are needed.

**Level of Evidence:**

Level I, systematic umbrella review of meta‐analyses on hip arthroscopy.

## Graphical abstract

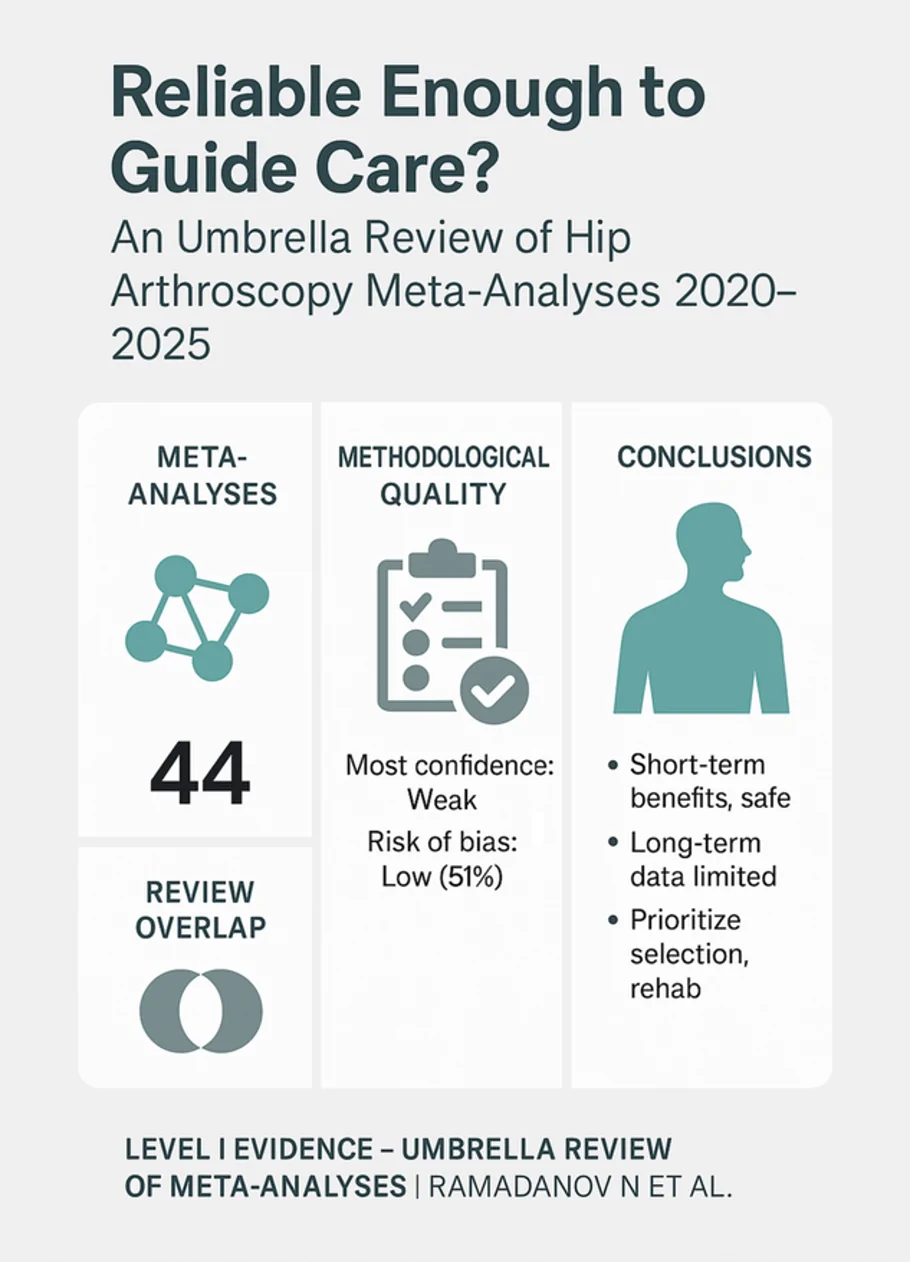

**Abbreviations**
CCA: corrected covered area
DOI: digital object identifier
FAI: femoroacetabular impingement
GRADE: grading of recommendations assessment, development and evaluation
HAS: hip arthroscopy
OCEBM: Oxford Centre for Evidence‐Based Medicine
OSF: Open Science Framework
PICOS: population, intervention, comparator, outcomes, study design
PMID: PubMed Identifier
PRIOR: Preferred Reporting Items for Overviews of Reviews
PRISMA: preferred reporting items for systematic reviews and meta‐analyses
PROMs: patient‐reported outcome measures
ROBIS: risk of bias in systematic reviews

## INTRODUCTION

Hip arthroscopy (HAS) has evolved from a niche diagnostic tool to a core hip‐preservation procedure for conditions such as femoroacetabular impingement syndrome (FAIS), labral tears and chondrolabral injury. Consensus guidance—the Warwick Agreement—formalised FAIS definitions (symptoms, clinical signs, imaging) and unified terminology, anchoring current indications and treatment principles [24]. Procedure volumes have risen steeply over the past two decades across health systems, driven by earlier diagnosis, improved instrumentation and streamlined perioperative care; large database studies show marked year‐on‐year growth [10, 16]. Because HAS now informs patient counselling and service planning, clinicians and healthcare decision‐makers need reliable syntheses to navigate a heterogeneous primary literature with variable indications, techniques, comparators and outcomes).

In evidence hierarchies, systematic reviews and meta‐analyses sit near the apex and provide structured methods to collate and weigh results across studies. The Oxford Centre for Evidence‐Based Medicine (OCEBM) levels are widely used to grade evidence and explicitly recognise rigorous systematic reviews in decision‐making [59]. Because meta‐analyses inform guidelines and clinical pathways, formal quality appraisal is essential. A MeaSurement Tool to Assess Systematic Reviews 2 (AMSTAR 2) offers a validated tool to judge methodological quality of meta‐analyses that include randomised and non‐randomised studies [78]. Complementarily, Risk Of Bias In Systematic reviews (ROBIS) assesses risk of bias at the review level—from study identification to synthesis—helping users distinguish reliable from potentially misleading summaries [86]. Another challenge is redundancy: multiple meta‐analyses often cover overlapping primary studies. Quantifying overlap (corrected covered area, CCA) and mapping concordance of conclusions help avoid double counting and clarify where findings converge [44, 69].

Against this backdrop, an umbrella review focused on HAS meta‐analyses can clarify what recent syntheses concludes, how trustworthy those conclusions are, and where gaps remain ‐ supporting transparent, evidence‐based recommendations for patient care and future research prioritisation.

This umbrella review aimed to synthesise meta‐analyses on HAS published from 1 January 2020 onward and to (i) identify eligible meta‐analyses, (ii) appraise methodological quality (AMSTAR 2) and risk of bias (ROBIS), (iii) quantify evidence overlap (CCA) and (iv) map concordance of conclusions.

## METHODS

### Protocol and registration

A protocol for this umbrella review was prospectively uploaded to the Open Science Framework (OSF; 28 September 2025; timestamped record). Reporting followed PRISMA (Preferred Reporting Items for Systematic Reviews and Meta‐Analyses) 2020 [63] and PRIOR (Preferred Reporting Items for Overviews of Reviews) guidance for overviews of systematic reviews [21].

### Eligibility criteria

We included systematic reviews with quantitative synthesis (meta‐analyses) on HAS in human patients, published 1 January 2020–1 October 2025. Eligible topics encompassed intra‐articular hip preservation indications (e.g., FAIS, labral pathology) and reported clinical effectiveness and/or safety (e.g., patient‐reported outcome measures [PROMs], pain, return to sport/work, complications, reoperation, THA conversion). We excluded systematic/narrative/scoping reviews without meta‐analysis, case series, technique notes, cadaveric/biomechanical studies, editorials, conference abstracts and retracted items. No language restrictions were applied.

### Information sources and search

We searched PubMed/MEDLINE, Embase and Epistemonikos from 1 January 2020, to 1 October 2025. The prespecified PubMed core string was: ((hip arthroscopy) OR (hip) OR (arthroscopy)) AND ((femoroacetabular impingement) OR (FAI)). This was combined with filters/terms for meta‐analyses (e.g., publication type: meta‐analysis, systematic review; title/abstract contains ‘meta‐analysis’, ‘systematic review’). Database‐specific adaptations were applied for other sources. Reference lists of included meta‐analyses were hand‐searched to identify additional eligible meta‐analyses. All records were de‐duplicated prior to screening. To maximise sensitivity, broad terms were used. Although terms such as ‘arthroscopic’ were not included as separate keywords, meta‐analyses on femoroacetabular impingement are consistently indexed under ‘hip arthroscopy,’ making missed eligible reviews unlikely.

### Study selection

Two reviewers (N.R. and M.V.) independently screened titles/abstracts and assessed full texts against prespecified eligibility criteria. At the title/abstract stage, records were retained if they mentioned HAS together with FAI syndrome or a closely related intra‐articular hip pathology (e.g., labral tear/chondrolabral injury). Disagreements were resolved by consensus or third‐reviewer (R.P.) adjudication. Reasons for full‐text exclusion were recorded, and study selection is summarised in a PRISMA 2020 flow diagram. At full text, we included systematic reviews with quantitative synthesis (meta‐analyses) reporting outcomes of HAS for FAIS or related conditions and excluded narrative/scoping reviews without meta‐analysis and studies not centred on HAS.

### Data extraction

Using a piloted form, two reviewers (N.R. and M.V.) in duplicate extracted: bibliographic data; clinical question/PICOS (population, intervention, comparator, outcomes, study design); meta‐analytic methods (databases, last search date, eligibility, risk‐of‐bias tools, random‐effects model/heterogeneity estimator, small‐study effects); number/type of primaries; pooled outcomes (effect direction/summary as reported); and authors' main conclusions. Primary data were not re‐pooled.

### Methodological quality and review‐level risk of bias

Methodological quality of each included meta‐analysis was appraised in duplicate with AMSTAR 2 (critical/non‐critical domains and overall confidence) [78]. Review‐level risk of bias was assessed with ROBIS (phases 2–3 domain judgments and overall risk) [86]. Methodological quality and review‐level risk of bias were assessed by two reviewers (N.R. and M.V.), while disagreements were reconciled by consensus.

### Evidence overlap and redundancy

We constructed a citation matrix (meta‐analyses × primary studies) and calculated the CCA [44, 69] to quantify overlap, categorising as slight <0.05, moderate 0.05–0.10, high 0.11–0.15, very high >0.15. Where multiple meta‐analyses addressed the same question with substantial overlap, we mapped concordance of conclusions (favour, no important difference, and inconclusive) and highlighted discordances.

### Synthesis and reporting

Findings were presented narratively and in structured evidence tables: topic, scope, AMSTAR 2 [78], ROBIS [86], CCA [44, 69] and conclusion concordance. Where meta‐analyses addressed comparable questions, we summarised the direction and credibility of effects without re‐meta‐analysing. Any deviations from the prespecified OSF protocol by the authors of this umbrella review were transparently reported.

## RESULTS

### Systematic review results

**Figure 1:**
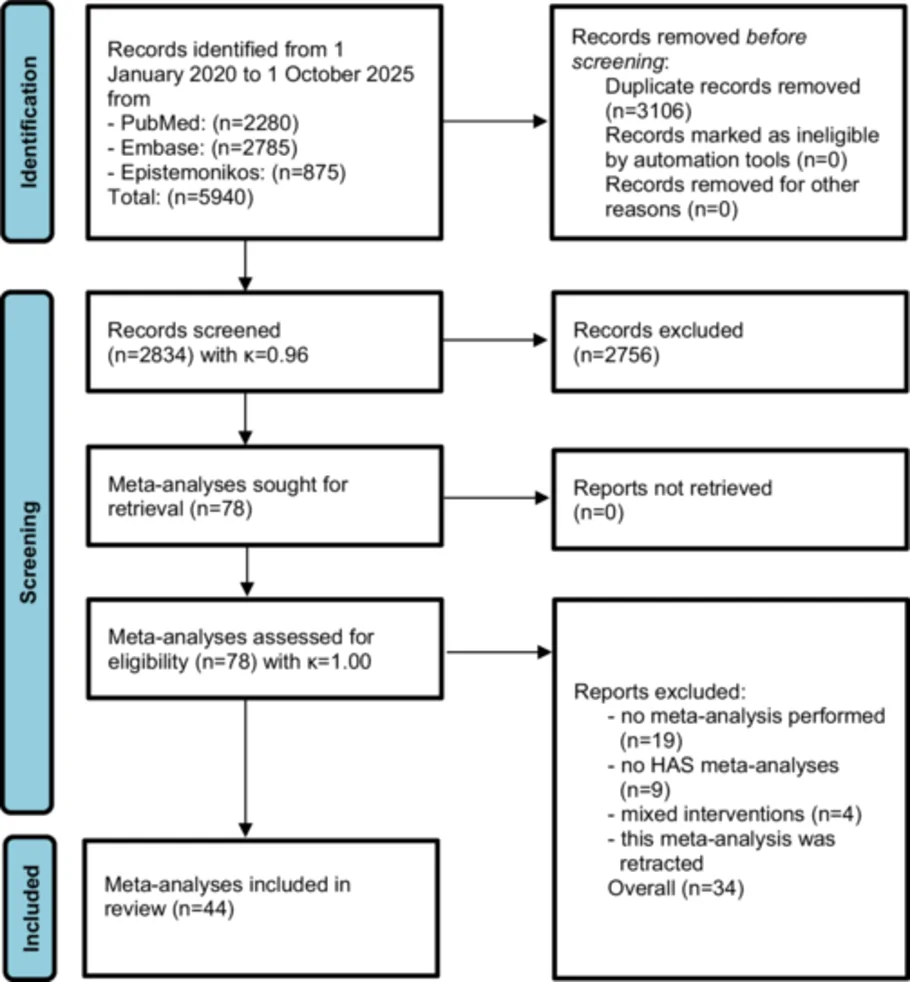
PRISMA flow diagram. HAS, hip arthroscopy; PRISMA, Preferred Reporting Items for Systematic Reviews and Meta‐Analyses.

The literature search yielded 5940 records. After removal of 3106 duplicates, 2834 records were excluded after title/abstract screening, leaving 78 records for full‐text assessment [1, 2, 3, 4, 5, 6, 7, 8, 9, 11, 12, 13, 14, 15, 17, 18, 19, 20, 22, 23, 25, 26, 27, 28, 29, 30, 31, 32, 33, 34, 35, 36, 37, 38, 39, 40, 41, 42, 43, 45, 46, 47, 48, 49, 50, 51, 52, 53, 54, 55, 56, 57, 58, 60, 61, 62, 64, 65, 66, 67, 68, 70, 71, 72, 73, 75, 76, 77, 79, 80, 81, 82, 83, 84, 85, 87, 88, 89]. After full‐text review, 34 records were excluded: (1) 19 systematic reviews without meta‐analysis [4, 5, 12, 27, 32, 34, 40, 43, 47, 49, 52, 53, 56, 62, 64, 76, 80, 82, 83]; (2) 9 not HAS intervention meta‐analyses (topic outside scope) [3, 13, 20, 28, 29, 67, 79, 81, 83]; (3) 4 mixed‐intervention reviews [23, 25, 55, 65, 88]; (4) one retracted record [37, 38]. Finally, 44 meta‐analyses were included in the present umbrella review [1, 2, 6, 7, 8, 9, 11, 14, 15, 17, 18, 19, 22, 26, 30, 31, 33, 35, 36, 39, 41, 42, 45, 46, 48, 50, 51, 54, 57, 58, 60, 61, 66, 68, 70, 71, 72, 73, 75, 77, 84, 85, 87, 89] (Figure 1).

### Description of the included meta‐analyses

Across 44 meta‐analyses (2020–2025) on HAS [1, 2, 6, 7, 8, 9, 11, 14, 15, 17, 18, 19, 22, 26, 30, 31, 33, 35, 36, 39, 41, 42, 45, 46, 48, 50, 51, 54, 57, 58, 60, 61, 66, 68, 70, 71, 72, 73, 75, 77, 84, 85, 87, 89], most addressed FAIS versus nonoperative care; others examined technique (capsular closure, labral repair vs debridement or reconstruction), HAS versus surgical dislocation (open), athlete outcomes, sex differences, revision HAS, femoral version subgroups and perioperative risk. Meta‐analyses typically pooled 3– >20 primary studies; the largest synthesised 74 studies (213,604 patients). Outcomes commonly included PROMs (iHOT‐33, HOS‐ADL/SS, mHHS, NAHS and HOOS), pain, MCID/PASS, revision/THA conversion, return to sport/work and complications. Random‐effects models predominated, but most meta‐analyses did not specify the between‐study heterogeneity estimator. When reported, DerSimonian–Laird and restricted maximum likelihood estimation (REML), both estimators of between‐study heterogeneity, were most common (Table 1).

**Table 1:** Characteristics of meta‐analyses on hip arthroscopy (HAS).

Study	Origin	Journal (ISSN)	Level of evidence	Studies included, *N*	Patients, *N*	Operative indication	Intervention	Comparator	Outcome parameters	Statistical paradigm (meta‐analytic framework)	Model type	Between‐study heterogeneity estimator	Remarks
Aamer et al. [1]	California Northstate University College of Medicine, Elk Grove, USA	*Cureus Journal of Medical Science (2168‐8184)*	Level 2	5	58,576	FAI	HAS with intra‐articular injections	HAS without intra‐articular injections	Postoperative infection rates, timing of injections	Frequentist meta‐analysis	REM	N.R.	Injection ≤3 months before HAS ↑ infection risk; >3 months appears safe
Addai et al. [2]	Department of Orthopaedic Surgery, University of Utah, Salt Lake City, USA	*The Bone & Joint Research Journal (2046‐3758)*	Level 2	48	4094	FAI	mini‐DAA, HAS	Surgical hip dislocation	HOS‐ ADL, HOS‐ SSS, mHHS, HOOS pain, HOOS symptoms HOOS ADL, HOOS sport and recreation, HOOS QoL, iHOT‐33, iHOT‐12, NAHS. Rates of complications, revision, and conversion to to tal hip arthroplasty. Change in α angle from pre‐ to postoperative	Frequentist meta‐analysis	REM and FEM	REML	AMO yields largest PROM gains but more complications; SHD ↑ THA risk
Assaf et al. [6]	Shamir Medical Center & Tel Aviv University, Israel	*Orthopaedic Journal of Sports Medicine (2325‐9671)*	Level 3	5	160	FAI	HAS	Preoperative baseline before HAS	Brake reaction time (BRT), total brake time (TBT), initial reaction time (IRT), sit‐to‐stand test (STST)	Frequentist meta‐analysis	REM	DerSimonian and Laird	Driving likely safe at 2–4 weeks (right) and ~2 weeks (left)
Bastos et al. [7]	Trata Institute Knee and Hip Rehabilitation, Brazil	*Clinical Rehabilitation (0269‐2155)*	Level 1a	3	650	FAI	HAS	Conservative treatment	isability (Hip Outcome Score, iHOT‐33), Adverse effects, Return to work	Frequentist meta‐analysis	N.R.	N.R.	Surgery ≈ conservative short/medium term; evidence moderate–low
Blaeser et al. [8]	NYU Langone Medical Center, New York University, New York, USA	*The American Journal of Sports Medicine (1552‐3365)*	Level 4	12	1124	FAI	HAS	None	Rate of return to work, Time to return to work	Frequentist meta‐analysis	N.R.	N.R.	Return to work ~71% in ~115 days; sedentary jobs fare better
Bolia et al. [9]	USC Epstein Family Center for Sports Medicine, Los Angeles, USA	*American Journal of Sports Medicine (0363‐5465)*	Level 4	29	1426	FAI	HAS	None	Return‐to‐sport rate, time to return to sport, intraoperative procedures	Frequentist meta‐analysis	REM and FEM	N.R.	Flexibility sports highest RTS; endurance fastest; publication bias possible
Casartelli et al. [11]	Schulthess Clinic & ETH Zurich, Switzerland	*Arthritis Care & Research (2151‐464X)*	Level 1	3	650	FAI	HAS	Conservative treatment	iHOT‐33 (hip pain, function, QoL)	Frequentist meta‐analysis	REM	N.R.	3 RCTs: higher short‐term iHOT‐33 after surgery versus PT
Cheong et al. [14]	Sengkang General Hospital, Singapore; Singapore General Hospital; Hospital for Special Surgery, NY, USA	*Journal of ISAKOS (2059‐7754)*	Level 4	6	498	FAI	HAS	None	mHHS, VAS, adverse events, revision surgery rate, conversion to total hip arthroplasty (THA)	Frequentist meta‐analysis	REM	N.R.	Asian cohorts: favourable PROs, low revision/THA rates
Cohen et al. [15]	McMaster University Medical Centre, Hamilton, Canada	*Knee Surgery, Sports Traumatology, Arthroscopy (0942‐2056)*	Level 4	36	2435	FAI	HAS	None	Radiographic outcomes (alpha angle, LCEA, Tonnis angle), functional outcomes (mHHS, iHOT, VAS), conversion to total hip arthroplasty (THA)	Frequentist meta‐analysis	REM	N.R.	No consensus targets; function improves despite variable correction
Dwyer et al. [17]	University of Toronto, Canada	*Arthroscopy (0749‐8063)*	Level 1	3	650	FAI	HAS	Conservative treatment	iHOT‐33, HOS, EQ‐5D, SF‐12, MCID, PASS	Frequentist meta‐analysis	REM and FEM	N.R.	Surgery > PT for iHOT‐33; more MCID/PASS after surgery
Elwood et al. [18]	University of Cambridge, Addenbrooke's Hospital, UK	*International Orthopaedics (0341‐2695)*	Level 4	22	1006	FAI	HAS	None	Return‐to‐play, time to return, PROMs (mHHS, NAHS, HOS, VAS), career length, further intervention rate	Frequentist meta‐analysis	REM	N.R.	Elites: ~94% RTS at ~7 months; ~10% reintervention
Ferreira et al. [19]	University of Sydney, Whitlam Orthopaedic Research Centre, Australia	*Journal of Science and Medicine in Sport (1440‐2440)*	Level 1	3	650	FAI	HAS	Conservative treatment	iHOT‐33, pain, range of motion, return to work, adverse events, cost‐effectiveness	Frequentist meta‐analysis	REM	N.R.	12 mo: surgery > non‐op; 24 mo: no clear advantage
Gatz et al. [22]	RWTH Aachen University Clinic, Germany	*European Journal of Orthopaedic Surgery & Traumatology (0942‐2056)*	Level 3	3	644	FAI	HAS	Conservative treatment	iHOT‐33 score, HOS (ADL & sport), EQ‐5D VAS, rate of osteoarthritis progression, rate of total hip arthroplasty	Frequentist meta‐analysis	REM and FEM	N.R.	Better iHOT/EQ‐VAS with surgery; THA similar
Huang et al. [26]	Peking University Third Hospital, Institute of Sports Medicine of Peking University, Beijing, China	*Clinical Journal of Sport Medicine(1050‐642X)*	Level 1	14	753	FAI	HAS	None	mHHS, HOS‐SSS, HOS‐ADL, NAHS, VAS pain, alpha angle correction, complication rate, revision rate	Frequentist meta‐analysis	REM	N.R.	Adolescents: large PRO gains, α − 22°, low reop
Kim et al. [30]	Gachon University Gil Medical Center, Korea	*Medicine (0025‐7974)*	Level 1	5	838	FAI	HAS	Conservative treatment	iHOT‐33, HOS, EQ‐VAS, mHHS, NAHS at 6 and 12 months	Frequentist meta‐analysis	REM	N.R.	At 12 mo: iHOT‐33 favours surgery; other scales mixed
King et al. [31]	La Trobe University, Melbourne, VIC, Australia	*Journal of Orthopaedic & Sports Physical Therapy (0190‐6011)*	Level 4	45	5500	FAI	HAS in female patients	HAS in male patients	Return‐to‐sport rate	Frequentist meta‐analysis	REM	DerSimonian and Laird	Females lower RTS odds, especially long‐term; very low‐certainty
Krivicich et al. [33]	Rush University Medical Center, Chicago, IL, USA	*Journal of the American Academy of Orthopaedic Surgeons (1067‐151X)*	Level 3	6	989	FAI	HAS in BDDH	HAS without dysplasia	HOS‐ADL, HOS‐SSS, mHHS, iHOT‐12, satisfaction, VAS pain	Frequentist meta‐analysis	REM	DerSimonian and Laird	Borderline dysplasia: short‐term PROs ≈ normal coverage
Kunze et al. [35]	Hospital for Special Surgery, New York, USA	*The Orthopaedic Journal of Sports Medicine (2325‐9639)*	Level 3	6	1611	FAI	HAS with capsular closure	HAS without capsular closure	Achievement of minimal clinically important difference (MCID) for mHHS, HOS‐ADL, HOS‐SS	Frequentist meta‐analysis	FEM	N.R.	Capsular closure ↑ MCID rates (small effect); trends on HOS
Lameire et al. [36]	University of Toronto, Canada	*The Orthopaedic Journal of Sports Medicine (2325‐9504)*	Level 4	16	2103	FAI	HAS	Conservative treatment	Radiographic progression of hip OA, conversion to total hip arthroplasty (THA), PROMs (mHHS, HOS, VAS)	Frequentist meta‐analysis	REM	N.R.	Possible ↓ OA progression vs non‐op; THA effect unclear
Lamo‐Espinosa et al. [39]	University Clinic of Navarra & Catholic University of Valencia, Spain	*Scientific Reports (2045‐2322)*	Level 3	6	839	FAI	HAS	Conservative treatment	iHOT‐33, HOS ADL, HOS Sports, adverse events, minimal clinically important difference (MCID)	Frequentist meta‐analysis	REM	N.R.	6 mo no difference; 12 mo iHOT/HOS‐ADL favour surgery
Lin et al. [41]	West China Hospital, Sichuan University, China	*HIP International (1798‐644X)*	Level 2	12	1185	FAI	HAS with capsular closure	HAS without capsular closure	Hip stability, patient‐reported outcomes (mHHS, HOS‐ADL, HOS‐SS), complications, revision rate, imaging findings (MRI, X‐ray)	Frequentist meta‐analysis	REM and FEM	N.R.	Early data: closure vs non‐closure similar PROMs/revisions
Liu et al. [42]	Department of Orthopaedics, The Third People's Hospital of Chengdu, China	*Journal of Orthopaedic Surgery and Research (1749‐799X)*	Level 1	11	902	FAI	HAS	Conservative treatment	Hip functional scores (mHHS, HOS), pain VAS), complication rates	Frequentist meta‐analysis	REM and FEM	N.R.	Mixed; some better HOS‐ADL without closure
Lv et al. [45]	Department of Orthopedics, The Second Affiliated Hospital of Guangzhou University of Chinese Medicine, Guangzhou, Guangdong, China	*Heliyon (2405‐8440)*	Level 4	15	2542	FAI	HAS with capsular closure	HAS without capsular closure	Surgery time, mHHS, HOS ‐ ADL & Sport, VAS, complication rate, revision rate, conversion to total hip arthroplasty (THA)	Frequentist meta‐analysis	REM and FEM	N.R.	Newer data: closure ↑ mHHS/HOS‐ADL, ↓ complications/THA
Mahmoud et al. [46]	Richmond, Australia	*Journal of Hip Preservation Surgery (2058‐8193)*	Level 1	4	749	FAI	HAS	Conservative treatment	Patient‐reported outcome measures (PROMs) including iHOT‐33, HOS ADL, EQ‐5D‐5L, SF‐12, Global Rating of Change (GRC), dGEMRIC (cartilage imaging)	Frequentist meta‐analysis	REM and FEM	REML	RCTs: surgery > targeted PT at 8–12 months
Marshall et al. [48]	University of Toronto, Sunnybrook Health Sciences Centre, St. Michael's Hospital, Toronto, Canada	*The Bone & Joint Journal (2049‐4394)*	Level 4	5	856	FAI	HAS	Conservative treatment	HOS ‐ ADL and Sport, iHOT‐33, VAS for pain, global rating of change (GRC), adverse events	Frequentist meta‐analysis	REM	N.R.	Biomechanics: selective improvements; very low‐quality evidence
Martins et al. [50]	Federal University of Santa Catarina; La Trobe University, Brazil	*Arthroscopy: The Journal of Arthroscopic and Related Surgery (1526‐3231)*	Level 3			FAI	HAS	None	Chondral damage severity	Frequentist meta‐analysis	REM	DerSimonian and Laird	Larger alpha angle → higher chondral damage risk; thresholds proposed
Migliorini et al. [51]	Departments of Life Sciences & Health Professions, Link Campus University of Rome, Italy	*Healthcare (2227‐9032)*	Level 4	3	808	FAI	HAS in athletes	HAS in non‐athletes	Patient‐reported outcomes: VAS, HOS, ADL, Sport‐Specific Subscale (SSS)	Frequentist meta‐analysis	REM and FEM	N.R.	RTS ~ 89%; better with younger age, lower weight, higher baseline scores
Migliorini et al. [54]	Department of Orthopaedic, Trauma, and Reconstructive Surgery, RWTH Aachen University Hospital, Aachen, Germany	*The Surgeon (2405‐5840)*	Level 3	41	4063	FAI	HAS	None	Return‐to‐sport rate; HOS‐SSS;	Frequentist meta‐analysis	REM	N.R.	Athletes vs non‐athletes: similar PROMs, reop, THA
Mok et al. [57]	First Affiliated Hospital of Jinan University & International School, Jinan University, Guangzhou; Pediatric Cardiac Surgery Center, National Center for Cardiovascular Disease and Fuwai Hospital, Beijing, China	*Orthopaedic Surgery (1755‐698X)*	Level 1	3	650	FAI	HAS	Conservative treatment	HOS, ADL, HOS sports, iHOT‐33	Frequentist meta‐analysis	REM and FEM	N.R.	Surgery > conservative for QoL/ADL; sports function NS
Ni et al. [58]	Department of Orthopedics, The Second Affiliated Hospital of Jiaxing University, Zhejiang, China	*BMC Musculoskeletal Disorders (1471‐2474)*	Level 3	9	1826	FAI	HAS	Open surgical dislocation	Alpha angle improvement, mHHS at 12 months; Nonarthritic Hip Score (NAHS) at 12 months; recurrence rate; complication rate	Frequentist meta‐analysis	REM and FEM	N.R.	HAS ≈ open for function; fewer recurrences; similar complications
O'Connor et al. [60]	Columbia University Irving Medical Center, New York, New York, USA	*American Journal of Sports Medicine (0363‐5465)*	Level 4	15	4316	failed FAI surgery	HAS	Revision HAS	mHHS, HOS‐ADL, HOS‐SSS, NAHS, VAS pain, SF‐12, revision rate, conversion to THA, complications	Frequentist meta‐analysis	REM	N.R.	Revision HAS improves PROs but less than primary; under‐resection common
Owen et al. [61]	Northwestern University Feinberg School of Medicine, Chicago, Illinois, USA	*The Orthopaedic Journal of Sports Medicine (2325‐9671)*	Level 3	74	213,604	FAI	HAS	None	Prevalence of cam, pincer, mixed impingement; femoroplasty, acetabuloplasty rates; mHHS, HOS‐ADL, HOS‐SS, iHOT‐12, VAS pain; complications; conversion to total hip arthroplasty (THA)	Frequentist meta‐analysis	REM and FEM	N.R.	Females more pincer, males more mixed; both exceed MCIDs
Patel et al. [66]	Robert Jones and Agnes Hunt Orthopaedic Hospital, Oswestry, UK	*HIP International (1724‐6067)*	Level 1	5	142	FAI	HAS	Preoperative baseline before HAS	BRT, IRT, TBT, foot movement time (FMT), brake pedal depression (BPD), throttle/turn reaction times (TRT/TTRT), gas‐off time (GOT), STST	Frequentist meta‐analysis	REM	DerSimonian and Laird	Driving typically safe by ~4 weeks post‐op
Phillips et al. [68]	Morristown Medical Center; Wake Forest University School of Medicine; Medical College of Georgia; University of Oxford; Orthopedic Institute (Sioux Falls), USA	*Arthroscopy: The Journal of Arthroscopic and Related Surgery (1526‐3231)*	Level 3	17	3191	FAI	HAS with capsular closure	HAS without capsular closure	complication rate	Frequentist meta‐analysis	REM	N.R.	Decision analysis: complete capsular closure yields higher ‘well’ outcomes
Qiao et al. [70]	Department of Orthopedics, The Second Hospital of Shanxi Medical University; Department of Joint and Sport Medicine, Tianjin Union Medical Center, China	*Medicine (0025‐7974)*	Level 3	7	606	FAI	HAS	Open surgical dislocation	mHHS, NAHS, VAS, satisfaction rate, postoperative alpha angle, internal rotation angle, complications	Frequentist meta‐analysis	REM and FEM	N.R.	Arthroscopy fewer complications; open better alpha/internal rotation change
Rahl et al. [71]	College of Human Medicine, Michigan State University, Grand Rapids, Michigan, USA	*The American Journal of Sports Medicine (0363‐5465)*	Level 3	8	537	Labral damage	HAS labral reconstruction with autograft	HAS labral reconstruction with allograft	conversion to total hip arthroplasty THA, complications, mHHS, HOS, ADL & SSS, NAHS, VAS, SF‐12), graft types	Frequentist meta‐analysis	REM	N.R.	Labral reconstruction improves PROs; allograft ≈ autograft
Ramadanov et al. [72]	Center of Orthopaedics and Traumatology, Brandenburg Medical School, Brandenburg an der Havel, Germany	*Bone & Joint Open (2633‐1462)*	Level 1	21	1799	FAI	HAS	Conservative treatment	HHS at 12 months, iHOT‐33 at 24 months, HOOS (Pain, Symptoms, ADL, Sports, QoL), HOS‐ADL, NRS, VAS, MCID for functional and pain parameters	Frequentist meta‐analysis	REM	REML	Multilevel RCT meta: higher HHS ≤ 12 mo and iHOT ≤24 mo with surgery; <MCID
Ramadanov et al. [73]	Center of Orthopaedics and Traumatology, Brandenburg Medical School, Brandenburg an der Havel, Germany	*Orthopaedic Surgery (1725‐1425)*	Level 1	7	973	FAI	HAS	Conservative treatment	HHS,MCID, iHOT‐33 at 12 months, HOS‐ADL at 8 months	Frequentist meta‐analysis	REM and FEM	Sidik–Jonkman with Hartung–Knapp adjustment	High‐quality meta: higher post‐op MCID and iHOT ≤12 mo for surgery
Rathore et al. [75]	Department of Trauma and Orthopaedics, Royal Berkshire Hospital, Reading, UK	*Hip & Pelvis (2287‐3279)*	Level 4	24	1619	FAI	HAS	None	mHHS, HOS‐ADL, HOS‐SSS, iHOT‐12, NAHS, VAS; Radiological parameters; Complications; Revision rates; Return to activity	Frequentist meta‐analysis	REM and FEM	DerSimonian and Laird	Adolescents: broad PRO improvements, few complications
Schwabe et al. [77]	Washington University School of Medicine, St. Louis, Missouri, USA	*The Orthopaedic Journal of Sports Medicine (2325‐9671)*	Level 1	3	650	FAI	HAS	Conservative treatment	iHOT‐33 HOS, ADL, HOS‐Sport	Frequentist meta‐analysis	REM	N.R.	3 RCTs: iHOT‐33 favours surgery; PT doesn't harm later surgery
Wang et al. [84]	Huashan Hospital, Fudan University, Shanghai, China	*The Orthopaedic Journal of Sports Medicine (2325‐9671)*	Level 4	5	822	FAI	HAS in abnormal femoral version	HAS in normal femoral version	mHHS,ΔmHHS, HOS‐SSS, NAHS, VAS pain, patient satisfaction, treatment failure	Frequentist meta‐analysis	REM	N.R.	Abnormal femoral version: outcomes ≈ normal version
Weber et al. [85]	University of Southern California, Los Angeles, California, USA	*The American Journal of Sports Medicine (0363‐5465)*	Level 3	20	1093	FAI	HAS	None	Rate of athletes failing to return to sport, reasons for nonreturn, level of competition, type of sport, subsequent hip surgeries (revision or total hip arthroplasty), patient characteristics	Frequentist meta‐analysis	REM	N.R.	Non‐RTS ~ 12%; mostly due to persistent hip pain
Wu et al. [87]	Shanxi Bethune Hospital & Shanxi Academy of Medical Sciences; Taiyuan Central Hospital; Tianjin Union Medical Center, China	*Medicine (0025‐7974)*	Level 2	5	323	FAI with labral tear	HAS with labral repair	HAS with labral debridement	mHHS, NAHS, VAS, satisfaction rate, surgical failure rate, complications	Frequentist meta‐analysis	REM and FEM	N.R.	Labral repair > debridement (mHHS, VAS, satisfaction)
Zhu et al. [89]	Department of Orthopedics, Orthopedic Research Institute, West China Hospital, Sichuan University, Chengdu, China	*Journal of Orthopaedic Surgery and Research (2052‐695X)*	Level 1	6	1187	FAI	HAS	Conservative treatment	iHOT‐33,HOS, VAS for pain, NAHS, EQ‐5D‐5L index, adverse events	Frequentist meta‐analysis	REM and FEM	N.R.	Surgery statistically > conservative on several PROMs (6–12 months)

Abbreviations: AA, alpha angle; ADL, activities of daily living; BHD/BDH, borderline dysplasia of the hip; BPD, brake pedal depression; BRT, brake reaction time; DAA, direct anterior approach; EQ‐5D, EuroQol 5 dimensions; EQ‐5D‐5L, EuroQol 5 dimensions, 5‐level; EQ‐VAS, EuroQol visual analogue scale; FAI, femoroacetabular impingement; FEM, fixed‐effects model; FMT, foot movement time; GOT, gas‐off time; HHS, harris hip score; HOOS, hip disability and osteoarthritis outcome score; HOOS‐ADL, HOOS‐activities of daily living; HOOS‐pain, HOOS‐pain; HOOS‐QoL, HOOS‐quality of life; HOOS‐sport, HOOS, sport and recreation; HOOS‐symptoms; HOOS, symptoms; HOS, hip outcome score; HOS‐ADL, hip outcome score‐activities of daily living; HOS‐SSS/SSS, hip outcome score‐sport‐specific subscale; iHOT‐12, international hip outcome tool‐12; iHOT‐33, international hip outcome tool‐33; IRT, initial reaction time; LCEA, lateral center‐edge angle; LD, labral debridement; LR, labral repair; MCID, minimal clinically important difference; mHHS, modified Harris hip score; NAHS, non‐arthritic hip score; N.R., not reported; NRS, numeric rating scale; OA, osteoarthritis; OR, odds ratio; PASS, patient acceptable symptom state; PRO, patient‐reported outcome; PROM, patient‐reported outcome measure; QoL, quality of life; RCT, randomised controlled trial; REM, random‐effects model; REML, restricted maximum likelihood (between‐study variance estimator); RR, risk ratio; RTS, return to sport; SF‐12, short form‐12; SHD, surgical hip dislocation; STST, sit‐to‐stand test; TBT, total brake time; TRT/TTRT, throttle/turn reaction times; THA, total hip arthroplasty; VAS, visual analogue scale.

### Methodological quality

**Figure 2:**
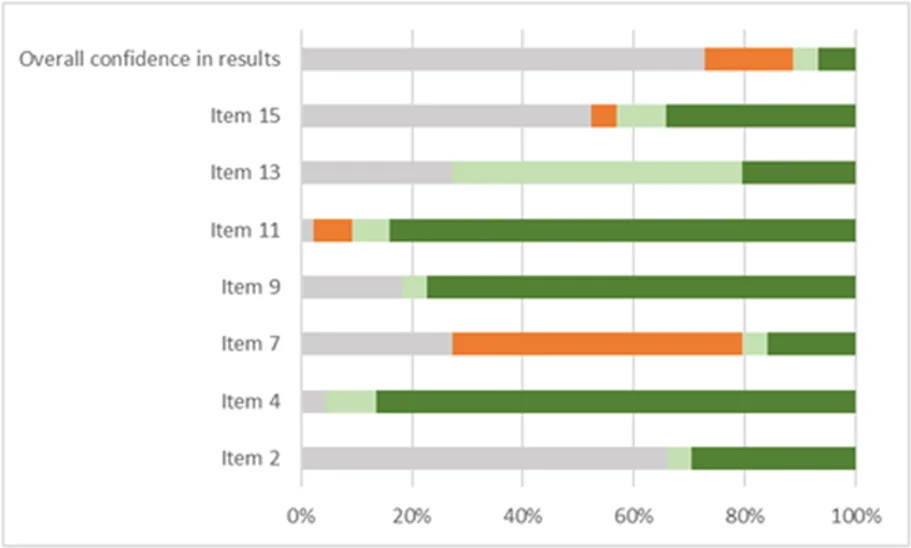
AMSTAR 2 summary across included meta‐analyses. Stacked 100% bars show the distribution of AMSTAR 2 judgments for the seven critical domains (Items 2, 4, 7, 9, 11, 13, 15) across 44 hip arthroscopy meta‐analyses, plus overall confidence. Colour key—items panel: grey = not reported, orange = no, light green = partially yes, dark green = yes. Overall confidence panel: grey = critically low, orange = low, light green = moderate, dark green = high. Visually, many reviews show No/Not reported for protocol registration (Item 2), a list/justification of excluded studies (Item 7), consideration of study risk of bias when interpreting results (Item 13), and assessment of small‐study/publication bias (Item 15). In contrast, domains on comprehensive search (Item 4), risk‐of‐bias assessment of included studies (Item 9) and appropriate meta‐analytic methods (Item 11) perform better. Overall confidence skews towards critically low/low, with relatively few moderate/high ratings.

Across the 44 meta‐analyses, methodological quality by AMSTAR 2 was predominantly weak: 3 (7%) high [31, 72, 73], 2 (5%) moderate [11, 26], 7 (16%) low [2, 7, 9, 19, 33, 39, 68] and 32 (73%) critically low [1, 6, 8, 14, 15, 17, 18, 22, 30, 35, 36, 41, 42, 45, 46, 48, 50, 51, 54, 57, 58, 60, 61, 66, 70, 71, 75, 77, 84, 85, 87, 89]. The most frequent critical shortcomings were absence of an a priori protocol/registration (Item 2; often NR), no list and justification of excluded studies (Item 7; often N/NR), not considering study risk of bias when interpreting results (Item 13; frequently PY/NR), and no assessment/discussion of publication bias (Item 15; often NR). In contrast, many meta‐analyses reported a comprehensive search (Item 4; mostly Y), assessed risk of bias of included studies (Item 9; often Y), and used appropriate meta‐analytic methods (Item 11; generally Y). Notably, only a small subset achieved high confidence (e.g., King et al. [31] Ramadanov et al. (1); [72] Ramadanov et al. (2) [73]), while a large majority were downgraded by one or more critical domains (Table 2, Figure 2).

**Table 2:** AMSTAR‐2 (condensed), adapted for systematic reviews with meta‐analyses.

Study	A priori protocol/registration (Item 2)	Comprehensive literature search (Item 4)	List and justification of excluded studies (Item 7)	Risk of bias of included studies assessed (Item 9)	Appropriate meta‐analytic methods (Item 11)	Consideration of RoB when interpreting results (Item 13)	Publication bias assessed and impact discussed (Item 15)	Overall confidence in results
Aamer et al. [1]	NR	Y	N	Y	N	PY	NR	Critically low
Addai et al. [2]	Y	Y	NR	Y	Y	PY	Y	Low
Assaf et al. [6]	Y	Y	NR	NR	PY	PY	NR	Critically low
Bastos et al. [7]	Y	Y	NR	Y	Y	Y	Y	Low
Blaeser et al. [8]	NR	PY	Y	PY	PY	PY	NR	Critically low
Bolia et al. [9]	NR	PY	Y	Y	Y	PY	Y	Low
Casartelli et al. [11]	PY	Y	Y	Y	Y	PY	PY	Moderate
Cheong et al. [14]	NR	Y	Y	Y	Y	NR	NR	Critically low
Cohen et al. [15]	NR	PY	NR	NR	PY	NR	NR	Critically low
Dwyer et al. [17]	NR	Y	N	Y	Y	PY	NR	Critically low
Elwood et al. [18]	Y	Y	N	Y	N	PY	NR	Critically low
Ferreira et al. [19]	Y	Y	N	Y	Y	Y	PY	Low
Gatz et al. [22]	NR	Y	N	Y	Y	PY	Y	Critically low
Huang et al. [26]	Y	Y	Y	Y	Y	PY	PY	Moderate
Kim et al. [30]	NR	Y	NR	NR	Y	NR	NR	Critically low
King et al. [31]	Y	Y	Y	Y	Y	Y	Y	High
Krivicich et al. [33]	Y	Y	Y	Y	Y	PY	NR	Low
Kunze et al. [35]	Y	Y	N	Y	Y	PY	NR	Critically low
Lameire et al. [36]	NR	Y	N	Y	Y	PY	NR	Critically low
Lamo‐Espinosa et al. [39]	Y	Y	NR	Y	Y	Y	Y	Low
Lin et al. [41]	NR	Y	NR	Y	Y	PY	N	Critically low
Liu et al. [42]	NR	Y	NR	Y	Y	PY	NR	Critically low
Lv et al. [45]	NR	Y	N	Y	Y	PY	NR	Critically low
Mahmoud et al. [46]	NR	Y	NR	Y	Y	PY	NR	Critically low
Marshall et al. [48]	NR	Y	NR	Y	Y	PY	NR	Critically low
Martins et al. [50]	PY	Y	N	Y	Y	Y	NR	Critically low
Migliorini et al. [51]	NR	Y	N	Y	Y	PY	NR	Critically low
Migliorini et al. [54]	NR	PY	N	NR	N	NR	NR	Critically low
Mok et al. [57]	NR	Y	NR	Y	Y	PY	NR	Critically low
Ni et al. [58]	NR	Y	NR	Y	Y	PY	NR	Critically low
O'Connor et al. [60]	NR	Y	N	PY	Y	PY	NR	Critically low
Owen et al. [61]	NR	Y	N	Y	Y	PY	NR	Critically low
Patel et al. [66]	NR	Y	N	Y	Y	Y	Y	Critically low
Phillips et al. [68]	Y	Y	N	Y	Y	Y	Y	Low
Qiao et al. [70]	NR	Y	N	Y	Y	NR	Y	Critically low
Rahl et al. [71]	NR	NR	N	NR	Y	NR	Y	Critically low
Ramadanov et al. [72]	Y	Y	PY	Y	Y	Y	Y	High
Ramadanov et al. [73]	Y	Y	PY	Y	Y	Y	Y	High
Rathore et al. [75]	NR	Y	N	Y	Y	NR	Y	Critically low
Schwabe et al. [77]	NR	Y	N	NR	NR	NR	NR	Critically low
Wang et al. [84]	NR	NR	N	NR	Y	NR	Y	Critically low
Weber et al. [85]	NR	Y	N	NR	Y	NR	Y	Critically low
Wu et al. [87]	NR	Y	N	Y	Y	NR	PY	Critically low
Zhu et al. [89]	NR	Y	N	Y	Y	NR	N	Critically low

*Note*: Columns: 7 critical domains (Y = yes; PY = partial yes; N = no; NR = not reported). Overall confidence (high/moderate/low/critically low) follows AMSTAR‐2 rules based on the number and nature of critical flaws.

Abbreviations: AMSTAR, A MeaSurement Tool to Assess Systematic Reviews; RoB, risk of bias.

### Review‐level risk of bias

**Figure 3:**
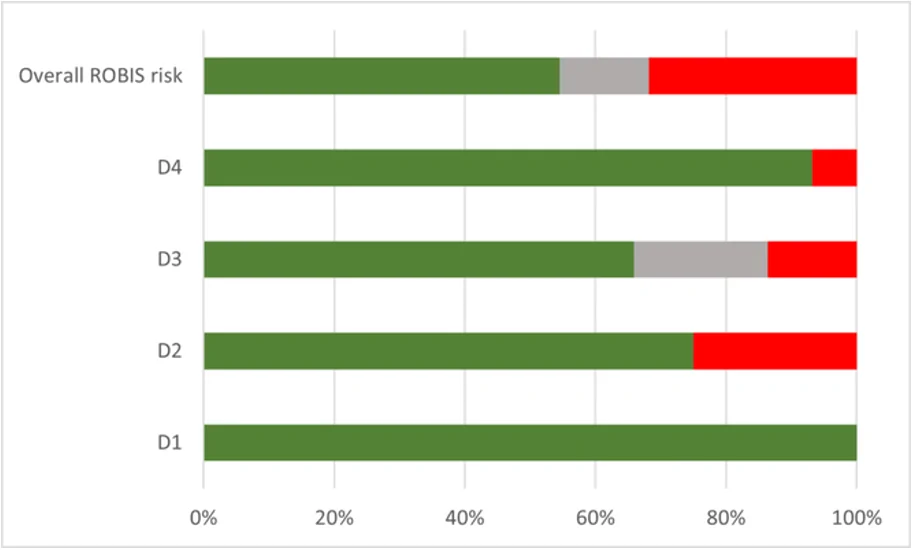
Risk of bias in systematic reviews (ROBIS) risk of bias by domain and overall. Stacked bars show the proportion of reviews rated low (green), high (red) and unclear (grey) for each ROBIS domain (D1–D4) and Overall. Overall, ROBIS was low in 24/44 (55%), high in 14/44 (32%) and unclear in 6/44 (14%). By domain, D1 (eligibility criteria) was uniformly low; D2 (identification/selection) showed the largest high component; D3 (data collection and appraisal) was predominantly low with some high/unclear ratings; and D4 (synthesis and findings) was mostly low with a smaller high segment.

Using ROBIS, 24/44 meta‐analyses were at overall low risk, 14/44 at high risk and 6/44 unclear. Domain‐level patterns were broadly consistent: D1 (eligibility criteria) was uniformly low risk. In contrast, D2 (identification/selection of studies) frequently showed high risk, reflecting concerns about search conduct and study selection. D3 (data collection and appraisal) was mostly low risk but included several high or unclear judgments where extraction methods, duplicate processes, or risk‐of‐bias assessments were insufficiently reported. D4 (synthesis and findings) was generally low risk, though a subset was rated high due to limitations in synthesis methods or interpretation. Overall, most meta‐analyses either met ROBIS standards or had clearly identifiable weaknesses concentrated in D2 and, to a lesser extent, D3/D4 (Table 3, Figure 3).

**Table 3:** Review‐level risk of bias (ROBIS).

Study	D1: Eligibility criteria	D2: Identification/selection	D3: Data collection and appraisal	D4: Synthesis and findings	Overall ROBIS risk
Aamer et al. [1]	Low	High	Low	High	High
Addai et al. [2]	Low	High	Low	Low	High
Assaf et al. [6]	Low	Low	Low	Low	Low
Bastos et al. [7]	Low	Low	Unclear	Low	Unclear
Blaeser et al. [8]	Low	High	High	High	High
Bolia et al. [9]	Low	High	High	High	High
Casartelli et al. [11]	Low	Low	Low	Low	Low
Cheong et al. [14]	Low	High	Low	Low	High
Cohen et al. [15]	Low	Low	Low	Low	Low
Dwyer et al. [17]	Low	High	Low	Low	High
Elwood et al. [18]	Low	High	Low	Low	High
Ferreira et al. [19]	Low	Low	Low	Low	Low
Gatz et al. [22]	Low	Low	Low	Low	Low
Huang et al. [26]	Low	Low	Low	Low	Low
Kim et al. [30]	Low	Low	Low	Low	Low
King et al. [31]	Low	Low	Low	Low	Low
Krivicich et al. [33]	Low	Low	Low	Low	Low
Kunze et al. [35]	Low	Low	Low	Low	Low
Lameire et al. [36]	Low	Low	Low	Low	Low
Lamo‐Espinosa et al. [39]	Low	Low	Low	Low	Low
Lin et al. [41]	Low	High	Unclear	Low	High
Liu et al. [42]	Low	High	Unclear	Low	High
Lv et al. [45]	Low	Low	Unclear	Low	Unclear
Mahmoud et al. [46]	Low	Low	Low	Low	Low
Marshall et al. [48]	Low	Low	Low	Low	Low
Martins et al. [50]	Low	Low	Low	Low	Low
Migliorini et al. [51]	Low	Low	Low	Low	Low
Migliorini et al. [54]	Low	Low	High	Low	High
Mok et al. [57]	Low	Low	Low	Low	Low
Ni et al. [58]	Low	Low	Unclear	Low	Unclear
O'Connor et al. [60]	Low	Low	High	Low	High
Owen et al. [61]	Low	High	High	Low	High
Patel et al. [66]	Low	Low	Unclear	Low	Unclear
Phillips et al. [68]	Low	Low	Low	Low	Low
Qiao et al. [70]	Low	Low	Unclear	Low	Unclear
Rahl et al. [71]	Low	High	Unclear	Low	High
Ramadanov et al. [72]	Low	Low	Low	Low	Low
Ramadanov et al. [73]	Low	Low	Low	Low	Low
Rathore et al. [75]	Low	Low	Unclear	Low	Unclear
Schwabe et al. [77]	Low	Low	Low	Low	Low
Wang et al. [84]	Low	Low	Low	Low	Low
Weber et al. [85]	Low	Low	Low	Low	Low
Wu et al. [87]	Low	Low	Low	Low	Low
Zhu et al. [89]	Low	Low	High	Low	High

*Note*: Overall ROBIS judgments were low in 24/44 (55%), high in 14/44 (32%) and unclear in 6/44 (14%). Domain patterns: D1 (Eligibility criteria) uniformly low risk; D2 (Identification/selection) frequently high risk; D3 (data collection and appraisal) mostly low with several high/unclear judgments; D4 (synthesis and findings) generally low with a subset high. Data are *n* (%).

Abbreviations: D1–D4, ROBIS domains; ROBIS, risk of bias in systematic reviews.

### Evidence overlap and redundancy

**Figure 4:**
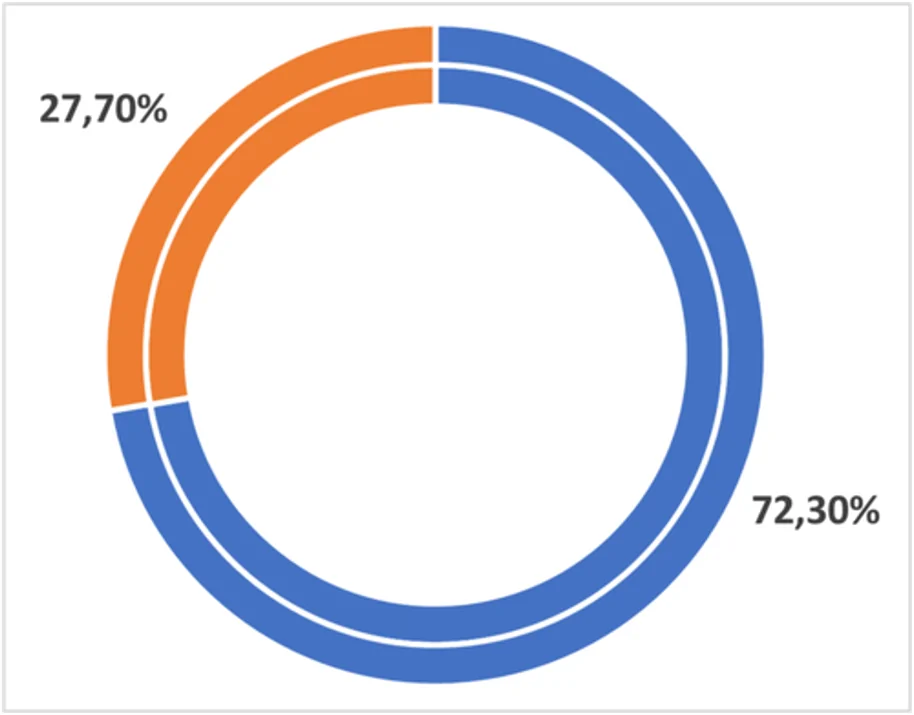
Evidence overlap across included meta‐analyses. (A) Corrected covered area (CCA) indicates slight overlap (CCA = 0.029). (B) A total of 448 unique primary studies (blue) contributed to 620 total study inclusions across 44 meta‐analyses. (C) Duplicate study inclusions (orange) totalled 172 (27.7%), yielding an average of 1.38 inclusions per primary study—consistent with minimal redundancy in the evidence base.

After de‐duplication by PMID/DOI, 448 unique primary studies contributed to 620 total study inclusions across 44 meta‐analyses. This resulted in a corrected covered area (CCA) of 0.029, indicating slight overlap (<0.05). Accordingly, each primary study contributed an average of 1.38 inclusions, consistent with minimal redundancy in the evidence base (Figure 4).

### Synthesis and reporting

Across randomised and comparative evidence, HAS provides superior short‐term improvement over best‐practice nonoperative care on hip‐specific PROMs—most consistently iHOT‐33 at ~8–12 months—with effects that attenuate by ~24 months and do not consistently reach established MCID thresholds as reported or applied within the included meta‐analyses across instruments [7, 11, 17, 19, 22, 30, 39, 46, 57, 72, 73, 77, 89]. Several RCT‐based syntheses (3–7 trials; *n* ≈ 500–1300) converge on superior iHOT‐33 at 8–12 months for surgery, while HOS‐ADL and other scales show smaller or inconsistent advantages, and intention‐to‐treat estimates are sensitive to crossover and heterogeneity in PT protocols [17, 19, 39, 46, 57, 77]. Compared with surgical dislocation (open), HAS shows similar functional gains with similar or fewer complications, albeit sometimes smaller alpha‐angle correction and internal rotation change [58, 70].

#### Return to activity

Return to work occurs in ~71% at a mean ~115 days, with full duty in ~51% and higher rates in less strenuous occupations [8]. Athletes generally fare well: elite cohorts return in ~94% at ~6–7 months with improved PROMs [18], and broader sport cohorts show ~89% return with higher postoperative activity linked to younger age, lower weight and better baseline HHS/NAHS/HOS‐ADL [51]. Sex/gender differences are notable: women have lower odds of return to sport at longer follow‐up (very low‐certainty evidence) [31], though both sexes exceed MCIDs post‐HAS and complication‐adjusted outcomes are broadly comparable [61]. Driving performance (brake reaction time) returns to baseline by 2–4 weeks (earlier for left‐sided procedures) [6, 66]. Athletes and non‐athletes demonstrate similar pain/function and reoperation at ~2 years [54]. In borderline dysplasia (BDH), isolated HAS achieves short‐term PROMs comparable to normal coverage, suggesting BDH itself is not a contraindication when carefully selected [33].

#### Technique questions

Evidence on capsular management is mixed over time: earlier comparative/meta‐analyses found no clear difference between closure and non‐closure [41, 42], whereas more recent, larger syntheses and decision‐analytic analyses favour closure, reporting slightly higher mHHS/HOS‐ADL and lower complications and THA conversion rates, at the cost of slightly longer operative time [35, 45, 68]. For labral treatment, repair generally outperforms debridement on mHHS, VAS and satisfaction with similar complications rates, supporting repair when feasible [87].

#### Structure–outcome and disease course

No consensus on an ‘optimal’ radiographic correction exists; functional gains appear consistent despite variable targets, with suggested postoperative means around alpha angle ~44° and LCEA ~ 30° [15]. Higher alpha angles predict more severe chondral damage (OR ≈ 1.04 per degree) independent of age and LCEA; men appear at higher risk overall [50]. Limited comparative data suggest HAS may reduce radiographic OA progression versus nonoperative care at long‐term follow‐up, without a clear signal for reduced THA conversion [36].

#### Safety

Preoperative intra‐articular injections ≤3 months of HAS are associated with increased postoperative infection risk, whereas injections >3 months before surgery show no increased risk [1]. Reported perioperative complications are generally low and lower with HAS than with open techniques [58, 70].

#### Context/limitations

Several syntheses highlight heterogeneity, possible publication bias and very low to moderate certainty for many outcomes, particularly beyond 12 months and for return‐to‐sport stratifications [9, 18, 31, 48, 61, 85]. Overall, the evidence supports HAS as an effective intervention for FAIS—especially for short‐term symptom and function improvement—with favourable safety and return‐to‐activity profiles, while longer‐term comparative benefits remain uncertain and likely depend on patient selection and technique.

## DISCUSSION

### Principal findings

This umbrella review synthesises 44 meta‐analyses on HAS published since 2020. Across randomised and comparative evidence, HAS provides superior short‐term improvement over best‐practice nonoperative care, most consistently for iHOT‐33 at ~8–12 months, with attenuation by ~24 months and inconsistent MCID attainment across instruments [7, 11, 17, 19, 22, 30, 39, 46, 57, 72, 73, 77, 89]. Compared with surgical dislocation (open), HAS achieves comparable functional gains with lower complication rates, despite occasionally less alpha‐angle correction and internal rotation change [58, 70]. Return‐to‐activity outcomes are favourable (~71% return to work by ~115 days; ~89%–94% return to sport), with possible sex‐based disparities in longer‐term return (very low‐certainty) [8, 18, 31, 51, 61]. Technique‐focused syntheses favour capsular closure for modest PROM advantages and fewer complications/THA conversion, whereas older and smaller meta‐analyses were neutral [35, 41, 42, 45, 68]. Labral repair outperforms debridement on several PROMs with similar safety [87]. Limited comparative data suggest possible reductions in radiographic OA progression versus nonoperative care without a clear THA‐conversion signal [36]. Intra‐articular injections ≤3 months before HAS are associated with increased infection risk, whereas injections >3 months show no increased risk [1]. No consensus optimal radiographic correction exists; proposed targets should be interpreted pragmatically [15]. Higher alpha angles predict more severe chondral damage independent of age/LCEA [50].

### How these findings fit with current concepts

The pattern of early symptomatic benefit with uncertainty about durability aligns with contemporary guidance emphasising that patient selection, structured rehabilitation and expectation management as pivotal. Signals favouring capsular closure, labral preservation and avoiding injections ≤3 months are consistent with evolving hip preservation principles that prioritise stability, seal restoration and infection mitigation [35, 45, 68, 87].

### Clinical implications

#### Counselling and timing

Patients can reasonably expect meaningful improvement by 8–12 months, particularly on iHOT‐33, with the caveat that benefits may attenuate beyond ~12–24 months and MCIDs are not consistently achieved across instruments [17, 19, 39, 46, 57, 77].

#### Nonoperative comparators

High‐quality physical therapy remains beneficial and does not compromise later surgical outcomes; it is an appropriate initial pathway in many cases [17, 46, 57, 77, 89].

#### Technique choices

When feasible, labral repair is supported by consistent evidence, and capsular closure by accumulating recent signals*—*for small but clinically relevant gains and lower complication/THA‐conversion rates [35, 45, 68, 87].

#### Perioperative safety

Avoid preoperative intra‐articular injection ≤3 months before surgery to reduce infection risk [1].

#### Special populations

BDH is not an absolute contraindication to isolated HAS if indications are strict and instability (and/or labral pathology)—and, where appropriate, labral pathology—is addressed [33]. Return‐to‐sport/work counselling should consider occupational demands and potential sex‐based differences in longer‐term participation [8, 18, 31, 51, 61].

### Methodological remarks and credibility

Methodological quality was predominantly weak by AMSTAR 2 (73% critically low), with common critical flaws (absent protocol/registration, missing exclusion lists, limited study‐ and publication‐bias considerations). Yet, ROBIS judged most meta‐analyses at overall low risk, with vulnerabilities concentrated in study identification/selection. Heterogeneity in physical therapy comparators, surgical era/technique, outcome timing and case‐mix likely explains the attenuation of pooled effects over time and the inconsistent MCID attainment.

### Statistical considerations

A common reporting gap was failure to specify the between‐study heterogeneity estimator. When reported, DerSimonian–Laird and REML predominated. Recent meta‐epidemiologic work [74] shows that estimator choice can meaningfully influence pooled effects and confidence intervals; in particular, DL without adjustment tends to yield too narrow intervals and more statistically significant findings in the presence of heterogeneity. Future HAS meta‐analyses should (i) explicitly report the estimator and any small‐sample or HK adjustments, (ii) pre‐specify and justify model choices and (iii) include sensitivity analyses across estimators to demonstrate result robustness.

### Redundancy and overlap

Despite 44 meta‐analyses, the CCA was 0.029 (slight), indicating relatively little reuse of primary studies across meta‐analyses. Topic fragmentation (e.g., capsular management, dysplasia, athletes, revision) likely dispersed overlap. Low redundancy reduces double‐counting concerns but disperses evidence, complicating cross‐topic inference.

### Research priorities

#### Durability and structure–function

Prospective, standardised follow‐up beyond 24 months to clarify sustained benefit, THA conversion and OA progression.

#### Comparator fidelity

Trials with protocolized, high‐quality physical therapy and transparent crossover/intention‐to‐treat handling to refine effect size and MCID interpretation.

#### Technique trials

Randomised/registry‐based comparisons of contemporary capsular strategies (preservation, (selective)closure/repair, non‐closure) and labral repair versus reconstruction with uniform PROMs/MCID/PASS reporting.

#### Equity and subgroups

Mechanistic and interventional studies to understand and mitigate sex‐based differences in return‐to‐sport; stratified analyses for borderline dysplasia, version abnormalities and high‐demand occupations [31, 33, 61, 84].

#### Safety

Prospective evaluation of injection timing, agent type and dose‐response on infection and outcomes [1].

#### Core outcome sets

Harmonise timing and instruments (iHOT‐33, HOS‐ADL/SS, mHHS and PASS/MCID) to reduce outcome reporting bias and facilitate meta‐research.

### Strengths and limitations of this umbrella review

Strengths include comprehensive capture of post‐2020 syntheses, dual ROBIS/AMSTAR‐2 appraisal and explicit overlap quantification. Limitations include reliance on reported (no repooling of) summary effects, heterogeneity of PT protocols and surgical eras across source meta‐analyses, and the predominance of very low to moderate certainty bodies of evidence in several domains.

## CONCLUSION

Across 44 meta‐analyses, HAS provides short‐term improvements over best‐practice nonoperative care, with benefits that attenuate by ~24 months and do not consistently meet MCIDs. Compared with open procedures, it offers similar functional gains with fewer complications. Technique‐focused evidence favours labral repair and, increasingly, capsular closure, preoperative intra‐articular injections within ≤3 months pre‐op should be avoided. Overall certainty is constrained by methodological limitations; careful patient selection, structured rehabilitation and transparent counselling on expected timelines remain essential, while higher‐quality, longer‐term trials are needed [1, 2, 6, 7, 8, 9, 11, 14, 15, 17, 18, 19, 22, 26, 30, 31, 33, 35, 36, 39, 41, 42, 45, 46, 48, 50, 51, 54, 57, 58, 60, 61, 66, 68, 70, 71, 72, 73, 75, 77, 84, 85, 87, 89].

## AUTHOR CONTRIBUTIONS

NR and MV conducted the systematic literature search, data extraction and methodological quality assessment. NR performed the statistical analyses and drafted the initial manuscript. All authors contributed to critical revision of the text, approved the final version, and agreed to be accountable for all aspects of the work.

## CONFLICT OF INTEREST STATEMENT

The authors declare no conflicts of interest.

## ETHICS STATEMENT

The authors have nothing to report.

## Supporting information

Citation matrix of primary studies across included meta‐analyses. Rows list primary studies; columns list meta‐analyses (n = 44). Column A shows the first author of each primary study; columns B–D report PMID, DOI, and publication year, respectively. In the ensuing 44 columns, each cell is coded “1” if that primary study was included in the corresponding meta‐analysis and left blank otherwise. This matrix was used to (i) identify unique primaries (n = 448), (ii) count total study‐inclusions across reviews (n = 620), (iii) quantify duplicate inclusions (n = 172; 27.7% of inclusions; mean re‐use 1.38 per primary), and (iv) calculate the Corrected Covered Area (CCA) for evidence overlap (CCA = 0.029; slight overlap). *Abbreviations: PMID, PubMed Identifier; DOI, Digital Object Identifier; CCA, Corrected Covered Area*.

## Data Availability

The authors have nothing to report.
